# Education and Consent for Population-Based DNA Screening: A Mixed-Methods Evaluation of the Early Check Newborn Screening Pilot Study

**DOI:** 10.3389/fgene.2022.891592

**Published:** 2022-05-12

**Authors:** Holly L. Peay, Angela You Gwaltney, Rebecca Moultrie, Heidi Cope, Beth Lincoln‐ Boyea, Katherine Ackerman Porter, Martin Duparc, Amir A. Alexander, Barbara B. Biesecker, Aminah Isiaq, Jennifer Check, Lisa Gehtland, Donald B. Bailey, Nancy M. P. King

**Affiliations:** ^1^ Genomics, Bioinformatics, and Translational Research Center, RTI International, Research Triangle Park, NC, Unites States; ^2^ Department of Biostatistics and Data Science, Wake Forest School of Medicine, Winston Salem, NC, United States; ^3^ Department of Pediatrics, Wake Forest School of Medicine, Winston-Salem, NC, United States; ^4^ Department of Social Sciences and Health Policy, Wake Forest School of Medicine, Winston-Salem, NC, United States

**Keywords:** informed consent, electronic consent, newborn screening, DNA screening, participant attitudes, evaluation

## Abstract

A challenge in implementing population-based DNA screening is providing sufficient information, that is, understandable and acceptable, and that supports informed decision making. Early Check is an expanded newborn screening study offered to mothers/guardians whose infants have standard newborn screening in North Carolina. We developed electronic education and consent to meet the objectives of feasibility, acceptability, trustworthiness, and supporting informed decisions. We used two methods to evaluate Early Check among mothers of participating infants who received normal results: an online survey and interviews conducted via telephone. Survey and interview domains included motivations for enrollment, acceptability of materials and processes, attitudes toward screening, knowledge recall, and trust. Quantitative analyses included descriptive statistics and assessment of factors associated with knowledge recall and trust. Qualitative data were coded, and an inductive approach was used to identify themes across interviews. Survey respondents (*n* = 1,823) rated the following as the most important reasons for enrolling their infants: finding out if the baby has the conditions screened (43.0%), and that no additional blood samples were required (20.1%). Interview respondents (*n* = 24) reported the value of early knowledge, early intervention, and ease of participation as motivators. Survey respondents rated the study information as having high utility for decision making (mean 4.7 to 4.8 out of 5) and 98.2% agreed that they had sufficient information. Knowledge recall was relatively high (71.8–92.5% correct), as was trust in Early Check information (96.2% strongly agree/agree). Attitudes about Early Check screening were positive (mean 0.1 to 0.6 on a scale of 0–4, with lower scores indicating more positive attitudes) and participants did not regret participation (e.g., 98.6% strongly agreed/agreed Early Check was the right decision). Interview respondents further reported positive attitudes about Early Check materials and processes. Early Check provides a model for education and consent in large-scale DNA screening. We found evidence of high acceptability, trustworthiness and knowledge recall, and positive attitudes among respondents. Population-targeted programs need to uphold practices that result in accessible information for those from diverse backgrounds. Additional research on those who do not select screening, although ethically and practically challenging, is important to inform population-based DNA screening practices.

## 1 Introduction

Precision public health implements DNA-based screening to identify individuals with specific characteristics and then target relevant interventions. Achieving the promise of equitable precision public health necessitates a basic understanding of genetic concepts among those offered DNA-based screening. Well-established challenges include the complexity of genetic and genomic information (Morgenstern et al., 2015) together with the relatively low health (Greenberg et al., 2007) and genomic literacy (Hurle et al., 2013) among U.S. residents.

Population-based DNA-based screening also creates feasibility challenges associated with scale. It is impractical for professionals to use traditional, face-to-face approaches to education and informed consent when implementing screening in public health and large-scale research settings. Electronic, user-driven approaches may improve practicability by alleviating professional and administrative burden, by making educational content more accessible to the target population, and through increasing the consistency of information provision. The development of end-user-focused education and informed consent procedures is critical to the success and feasibility of public health integration of genetics and genomics.

Early Check is a voluntary, large-scale expanded newborn screening (NBS) research study in North Carolina, established to address substantial gaps in newborn screening evidence and to inform policy (Bailey et al., 2019). The study is led by researchers at RTI International, in partnership with the University of North Carolina at Chapel Hill, the North Carolina State Laboratory of Public Health (NCSLPH), Duke University, and Atrium Health Wake Forest Baptist (formerly Wake Forest Baptist Medical Center). Early Check offers new and expectant mothers or legal guardians screening for conditions that are not currently included in state NBS; the Early Check panel has included spinal muscular atrophy (SMA), fragile X syndrome (FXS), and Duchenne muscular dystrophy (DMD). Early Check currently does not use sequencing in the initial screening. Targeted genetic analysis was used for SMA and FXS, and creatine kinase isoenzyme (CK-MM) was used for DMD screening.

Babies who receive NBS through the NCSLPH and live in North or South Carolina are eligible for participation in Early Check. Mothers or legal guardians can enroll if they are at least 13 weeks pregnant or have a baby up to 4 weeks of age. All mothers or legal guardians who have given birth in North Carolina and whose babies have newborn screening are mailed an invitation letter and flyer from the NCSLPH. Collaboration with partners at University of North Carolina at Chapel Hill and Duke University supports in-person recruitment at those affiliated birthing hospitals and prenatal invitations sent via MyChart. Early Check also has a social media presence via Facebook, Twitter, and Pinterest.

The research screening is done using residual dried blood spots obtained for standard NBS and retained by the NCSLPH (North Carolina Department of Health and Human Services, 2020). The Institutional Review Board at the University of North Carolina at Chapel Hill determined that the Early Check study is minimal risk; thus, only the mother is required to give permission for the child to participate, though the study materials encourage both parents to be involved in the decision making, as relevant. Because traditional education and consent approaches are impracticable given the approximately 1,20,000 births per year in North Carolina, the study team developed a user-driven, participant-centered digital education and electronic consent approach. Our development objectives were:

• Feasibility for the research team;
• Acceptability and trustworthiness for potential participants; and
• Supportive of informed decision-making.

Electronic consent refers to the use of digital means to obtain informed consent from potential study participants. The U.S. Food and Drug Administration (2015) defines this as “the use of electronic systems and processes that may employ multiple electronic media, including text, graphics, audio, video, podcasts, passive, and interactive Web sites …. to convey information related to the study and to obtain and document informed consent.” Electronic consent may enhance knowledge and engagement of study participants in comparison to traditional informed consent, and improve quality and consistency of the consent process (Rowbotham et al., 2013; Rothwell et al., 2014; Simon et al., 2016; Cadigan et al., 2017; Buckley et al., 2018; Biesecker et al., 2019). Additionally, electronic consent leverages digital tools to improve visual clarity and focus on content most important to decision making and reduces the length, complexity, and literacy demand of consent materials. Such approaches may be more engaging, participant-centered, and help address long-reported issues with standard informed consent (Biesecker et al., 2019; Grant, 2021).

Early Check’s approach was created by a multidisciplinary team that included experts in health communication, informed consent, clinical genetics, behavioral science, user interface development, and bioethics. We employed user-centered design that integrated community engagement and rounds of formative research with diverse participants. The resulting electronic consent includes 16 screens with core information presented in lay language, and which offer additional detail in layered (optional) content. The electronic consent includes an interactive eligibility tool and employs simple graphics, infographics, and videos. The content provides a brief values clarification that provides reasons a mother might participate or decline. It concludes with summarizing self-assessment questions. All screens include optional voiceover to reduce literacy demands, options for contacting the study team, and a list of the collaborating institutions. The electronic consent sections and a brief description of section components (in addition to standard text elements) are described in Table 1.

**TABLE 1:** Early Check electronic consent overview.

Section title	Components in addition to standard text
Welcome to Early Check! Let’s get started!	Video; Eligibility screener; Visual overview of e-consent process
How is Early Check done?	Video; Infographic
What health problems does Early Check look for in newborns?	Learn more about [condition name] from our experts
What happens when parents get results from Early Check?	Information for parents of twins or multiple babies
Do you have to pay for Early Check?	
How is Early Check different from state newborn screening?	Learn more about regular North Carolina newborn screening from our experts
Are the screening tests perfect?	Learn more about Early Check’s false positive rates; Learn more about screening tests from our experts
How is your information protected and shared?	Learn more about protecting information from our experts
Why might you say Yes to Early Check? And why might you say No?	Video; Interactive checklist
Let’s Review	Review questions, multiple choice format with correct responses shown and explained
Agreement and electronic signature	Option to continue to electronic signature page, or take more time to decide (with option to enter email address to receive a reminder) or to contact study team with questions

All materials are available in English and Spanish. We developed the education and consent process so that it does not require investigator involvement unless clarification or assistance is requested by a parent. A copy of the Early Check e-consent content is available for reader review: https://testportal.earlycheck.org/. Here we present results from an evaluation of the Early Check electronic education and consent.

## 2 Materials and Methods

We implemented a mixed-methods evaluation using data from mothers or legal guardians who enrolled their newborns in Early Check. Our survey aims were to assess, among mothers who chose to enroll their child and received a normal result:

• Motivation for enrolling the child in Early Check,
• Whether the process was acceptable and information sufficient,
• Attitudes about Early Check screening and participation in the research,
• Knowledge recall of key facts about Early Check, and
• The degree to which Early Check was perceived as trustworthy.

For knowledge recall and trust, an additional aim was to determine whether there were differences based on race/ethnicity and educational attainment. We also tested our hypothesis that trust ratings would be higher in those who rated themselves as sufficiently informed to make the decision to enroll in Early Check, those with more positive attitudes toward screening, and those with higher knowledge recall.

The evaluation also included semi-structured interviews with mothers of infants enrolled in Early Check to explore similar concepts in more depth and to allow for the emergence of unexpected attitudes or experiences with the study.

### 2.1 Inclusion and Recruitment

Between 7/7/2020 and 11/17/2021, mothers aged 18 or older whose child received a normal Early Check screening result were invited to participate in the evaluation survey. Interviews were conducted between 7/13/2020 and 8/31/2020 with mothers who met the same criteria. These evaluation efforts were directed to mothers of children with normal results. We are also conducting mixed-methods research, which is still underway and will be reported separately, on parents whose children received an abnormal, actionable result. Given the different experience and level of engagement that families of screen positive infants have with Early Check, the assessment of parents whose children receive an abnormal result is conducted using a longitudinal, mixed methods approach, with greater depth to the questioning about the impact of the study result.

Participants were recruited via email and the Early Check return of results website. Those who completed the survey were entered in a monthly drawing to receive a $20 gift card, and all interview participants received a $20 gift card.

The evaluation activities were approved by the University of North Carolina at Chapel Hill Institutional Review Board as a modification to the overall Early Check study (#18–0009) ***.***

### 2.2 Evaluation Survey

The evaluation survey was a 36-question questionnaire conducted online. The survey instrument included the following constructs and demographic questions.

#### 2.2.1 Motivations for Enrolling the Baby in Early Check

Respondents were asked to select the reasons they enrolled their baby in Early Check, using response options informed by the consent information and prior formative research (Peay et al., 2018). Respondents first chose up to three responses from the following options: “It was free,” “To help babies in the future,” “It was easy to sign up,” “It did not require a doctor visit,” “There were not additional blood samples taken from my baby,” “To find out if my baby has the conditions screened,” “For my peace of mind,” “To help research,” “I don’t recall,” and “Other.” They were then asked to select the single most important reason from the three they initially selected.

#### 2.2.2 Acceptability and Sufficiency of Information in the Enrollment Process

Respondents’ preference for learning about and signing up for Early Check was assessed with a single ranking item, with options that included, “get information about Early Check online and sign up on my own”, “Get information from a healthcare provider/health educator and also get information about Early Check online and sign up on my own”, and “Get information from a healthcare provider/health educator and sign up with them”.

Respondents answered three questions about Early Check information using a 5-point rating scale ranging from not at all to a good amount. The items were “Did the Early Check information make it easier to make a decision about whether to sign up?”; “How helpful was the information provided by Early Check in making the decision to sign up?”; and “How much did the information about Early Check help you understand what you were signing up for?”

Respondents were then asked a yes/no question, “Did you get enough information about Early Check?” If respondents marked that they did not get enough information, they were asked a follow-up question to indicate what more they hoped to learn, with items including “More about the conditions screened,” “More about the Early Check process,” “More about newborn screening,” “More about my child’s participation and expectations,” or “Other.” Respondents were then asked (yes/no), “With the same information you got, do you think other parents will be able to make a decision about signing up for Early Check?”

#### 2.2.3 Attitudes About Early Check Screening and Participation

We included five items on attitudes toward the screening, using items originally from Marteau et al. (2001), as adapted by Lewis and colleagues (2016). Respondents marked their answers to semantic differential items anchored by opposite descriptors, with response options ranging from 0 to 4: “For me, having Early Check was…beneficial/harmful, important/unimportant, a good thing/a bad thing, reassuring/not reassuring, and desirable/undesirable” (Lewis et al., 2016). We selected three items from the Decision Regret Scale (O’Connor et al., 2003) that are relevant to the decision context: “It was the right decision,” “I regret the choice that was made,” and “I would go for the same choice if I had to do it over again.” Response options were on a 5-item Likert-type response ranging from strongly agree to strongly disagree.

#### 2.2.4 Knowledge Recall About Early Check

We included a series of six questions to assess knowledge recall of Early Check concepts. Response options were True/False/Unsure. Respondents marked the answers to the following questions (correct response noted in parenthesis):

• Early Check screening tests will not find every single baby with the health problems. (True)
• If the screening result is not normal that means the baby definitely has the health problem. (False)
• Early Check screens for health problems that currently cannot be cured. (True)
• Early Check does the test on the same blood spot taken from the baby’s foot after delivery. (True)
• There are treatments that can help babies with the health problems screened by Early Check. (True)
• Finding health problems early gives babies a chance for better development and health outcomes. (True)

#### 2.2.5 Trust in the Information Provided About the Early Check Study

Respondents were queried about how much they agreed or disagreed with the statement “I trust the information provided by Early Check.” Response options were on a 5-point scale from strongly agree to strongly disagree.

The survey included additional questions related to condition familiarity and perspectives on the return of results process, which are not included in this analysis.

#### 2.2.6 Analysis

Statistical analysis was performed using SAS version 7.15. Descriptive statistics were used to characterize participant demographics. Chi-square and t-tests were completed to assess differences in participant characteristics between mothers who completed the survey (using race, ethnicity and education data provided in the survey) and the population of mothers who enrolled their infants in Early Check during the same time period but did not complete the survey (using race, ethnicity and education data provided at the time of enrolling the infant in Early Check).

Descriptive analysis was used to summarize responses to the survey items. Several planned analyses to assess factors associated with acceptability and participant attitudes could not be conducted because of highly skewed data.

Knowledge recall items were summed, based on scoring a one for a correct response and 0 for an incorrect or uncertain response, resulting in a range of 0–6. An unadjusted, ordered logistic regression was used to determine whether there were significant differences in knowledge recall scores between White and non-White participants; between Hispanic/Latino and non-Hispanic/Latino participants; among those with less than a bachelor’s degree, a bachelor’s degree, or more than a bachelor’s degree; and based on participant age. Those who did not provide race or ethnicity were removed from this analysis. An adjusted model with all significant characteristics was then conducted.

For trust, we dichotomized the dataset into those who strongly agreed/agreed with trusting Early Check versus those who were unsure, disagreed, or strongly disagreed. We then applied univariate statistical analysis (Chi-Square or Fisher’s exact test for categorical, Kruskal-Wallis test for ordinal variables, and Wilcoxon-Mann-Whitney U test for interval data) to assess differences among the groups based on their race, education, mean attitude score about Early Check screening, knowledge recall score, and whether they perceived themselves to be sufficiently informed (yes/no). Output from the Wilcoxon-Mann-Whitney U tests results were used to display box plots of differences in Wilcoxon mean scores by trust category.

### 2.3 Semistructured Interviews

The evaluation interviews were conducted *via* telephone. Interviews were conducted by an experienced qualitative researcher from Wake Forest School of Medicine who was not involved in the day-to-day operations of the study. Interviews lasted between 20 and 30 min.

The interviewer used a semi-structured interview guide. Interview questions were designed to explore similar evaluation constructs as the survey. Domains included motivations for enrollment, perception of information sufficiency ease of using the Early Check electronic consent process, perceptions of trust, and satisfaction with the decision to enroll their infant. Data on mothers’ age, race, ethnicity and educational attainment were obtained at the time of enrollment of the infant in Early Check.

#### 2.3.1 Analysis

Interviews were recorded and transcribed verbatim for analysis. Two experienced coders from RTI who were not involved in the planning or conduct of the Early Check study iteratively coded all interview transcripts using *in vivo*. A codebook was first developed with inductive and deductive codes to organize and label the interview data. Coders then selected four interviews to code simultaneously to establish interrater reliability using Cohen’s κ. Strong agreement was found between the two coders, κ = 0.92. An inductive approach was used to analyze the data and identify themes across interviews. Excerpts from verbatim transcripts were selected to illustrate themes.

## 3 Results

### 3.1 Participant Characteristics

Of 1,837 survey respondents meeting study criteria (a 24% response rate), most remembered giving permission for their babies to be enrolled in the Early Check study (*n* = 1,823). Six respondents (0.003%) did not remember and eight (0.004%) who were unsure were excluded from the following analysis.

Of the resulting 1,823 respondents, 69% were White, 6% Black, 6% Asian, 15% missing race/preferred not to answer, and 9% were Hispanic/Latino. Seventy-four percent of survey respondents had a bachelor’s degree or higher (Table 2). In contrast, the North Carolina population is approximately 60% White, 12% Black, and 6% Asian; and 10% Hispanic/Latino. Approximately 30% of the North Carolina population have a bachelor’s degree or higher (U.S. Census, 2018; U.S. Census, 2020).

**TABLE 2:** Characteristics of parents who enrolled their infants in Early Check and received negative screening results, survey respondents, and interview participants.

	Parents who enrolled infant in EC (*n* = 7,702) 7/7/2020–11/17/2021	Survey respondents (*n* = 1,823) 7/7/2020–11/17/2021	Interviewees (*n* = 24) 7/13/2020–8/31/2020
Median age (years)	32 (11–51)*	33 (18–46)**	35 (23–41)
Ethnicity			
Hispanic or Latino	1,067 (14%)	159 (9%)	2 (8%)
Not Hispanic or Latino	6,092 (79%)	1,395 (77%)	20 (83%)
Unknown/Not reported	543 (7%)	269 (14%)	2 (8%)
Race			
White	5,446 (71%)	1,250 (69%)	18 (75%)
African American/Black	691 (9%)	118 (6%)	4 (17%)
Asian	512 (7%)	104 (6%)	2 (8%)
American Indian/Alaska Native	36 (0.5%)	4 (0.2%)	0
Multi-race/Other	751 (9%)	66 (4%)	0
Unknown/Not reported	266 (4%)	281 (15%)	0
Education			
Did not finish high school	30 (0.4%)	18 (1%)	0
High school graduate	53 (0.7%)	109 (6%)	0
Some college	73 (1%)	123 (7%)	1 (4%)
College degree or higher	468 (6%)	1,343 (74%)	2 (8%)
Not reported	7,078 (92%)	232 (13%)	21 (88%)

*Those with reported maternal ages greater than 60 (n = *3*) were excluded because of anticipated data entry error.

**Derived from 983 participants with completion dates available to calculate age.

Twenty-four mothers participated in the in-depth interviews. Seventy-five percent of interviewees reported their race as White, 17% as Black, and 8% Hispanic or Latino. Four percent reported some college experience and 8% a college degree, although the majority (88%) preferred not to report their education.

Comparing survey respondents to mothers of all Early Check participants who were recruited during the same time period but did not complete the survey (*n* = 7,702), there were significant differences in age (*t* (7,729.6) = 1,051.19, *p* < 0.0001), ethnicity [*X*_2_ (2, *n* = 9,525) = 134.1, *p* < 0.0001], and race [*X*_2_ (5, *p* < 0.0001], although the differences were modest. The amount of missing data about maternal education precluded education-based comparisons.

The sample size of interviewees was too small to make statistical comparisons. Table 2 includes demographic data provided by mothers when they enrolled their infants in the Early Check study.

### 3.2 Motivations for Enrolling the Baby in Early Check

#### 3.2.1 Evaluation Survey

**FIGURE 1:**
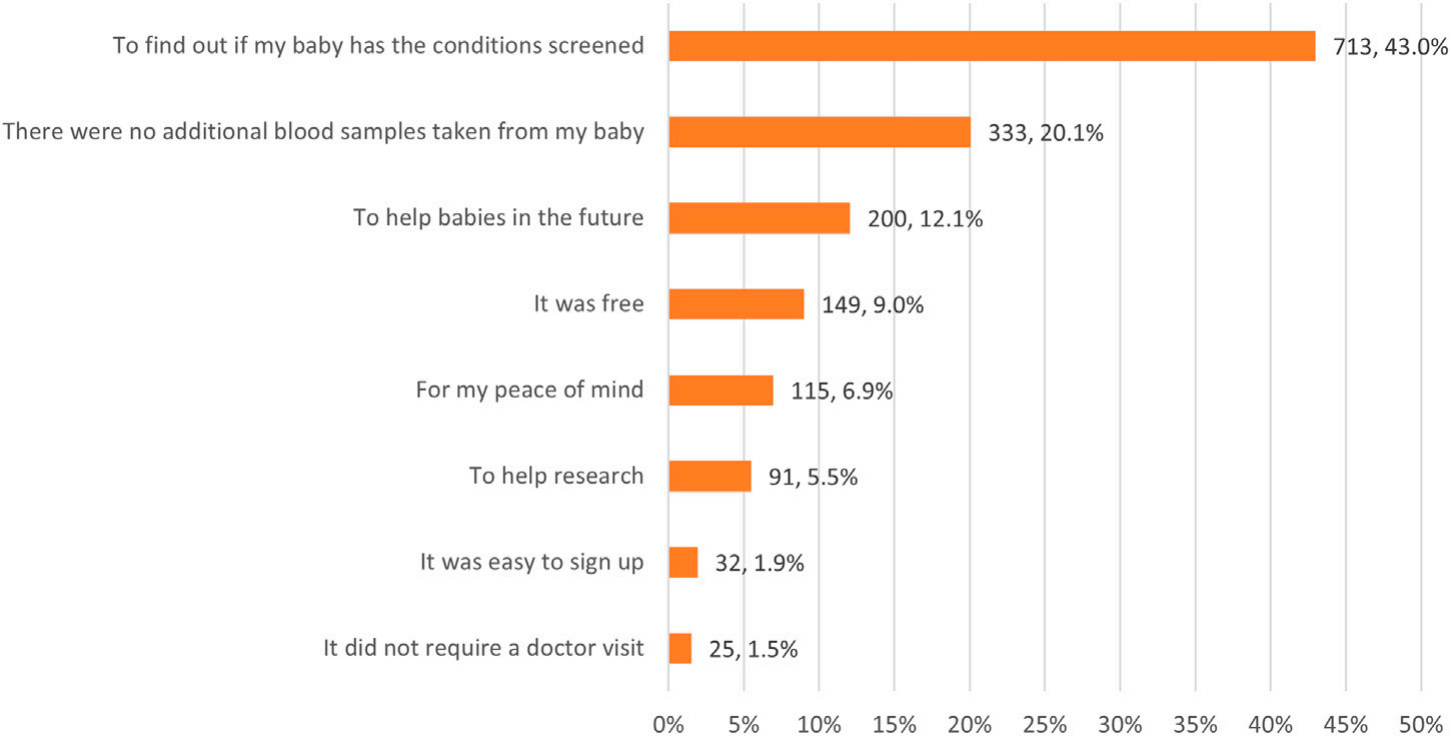
Most important reasons for enrolling the baby in Early Check (*n* = 1,665).

The most frequently-endorsed reason for enrolling was to find out if the baby has the conditions screened (43.0%), followed by the need for no additional blood samples from their baby (20.1%) (see Figure 1).

#### 3.2.2 Interviews

All but one interviewee reported that a main reason for signing up was to know if their child had one of the conditions screened. They indicated wanting to be armed with information, and many expressed the sentiment of, “I would rather know than not know.” Many also reported that they thought getting normal results would give them peace of mind.

“It seemed like a nice opportunity to learn more about our child potentially—like obviously if there is a genetic condition that we were not already aware of, it would be nice to know.”

“I was interested [in] her [getting] screened for everything she possibly could. So, I could just clear my mind of any existing problems that she might have.”

Many interviewees shared that knowing about the conditions early would allow them to be prepared and to seek necessary resources or treatment for their child.

“The more screening you can do to understand your child and how you can help them, the better…. the more that you can see coming, the better prepared you are—if you know about it, then you can help them be prepared with early treatment.”

A few noted specific reasons to be concerned about the health of their babies because of a high-risk pregnancy or a family history of one of the genetic disorders.

“I’m a high-risk patient, so like anything that would give me a better insight towards anything that might affect my baby…. Basically, I would take the answers.”

Ease of participation motivated enrollment among interviewees.

“I read through the information and figured there was nothing to lose, so it’s not like we had to do a whole bunch on our part. It was…signing up online and allowing his blood, or whatever it was, to get used from the hospital. So, it’s not like we had to go in and do anything extra… I’m quite sure if we did have to go back to the hospital or something—I’m sure I wouldn’t have done it. But it was easy enough just to use what the hospital already had.”

Several interviewees reported that they wanted to contribute to research and viewed the program as a way of helping other families or children.

“In general, just having the information for ourselves and if we needed to do anything further, and then just helping out others to be able to have that information as well.”

Participants were asked if they had any concerns when signing up for Early Check. Most respondents shared that they had no concerns. A few had concerns related to the privacy of their child’s genetic information.

“We had the slightest, slightest hesitation in thinking the only possible downside of this is that now like the state has our child’s genetic material and she’s like an infant, right?…. I don’t think they’re going to do anything weird with our information. It is obviously all confidential.… So that was just like the slightest little hesitation, but we don’t think that there’s anything negative that will come out of it in that way, really.”

### 3.3 Acceptability and Sufficiency of the Early Check Enrollment Process

#### 3.3.1 Evaluation Survey

**FIGURE 2:**
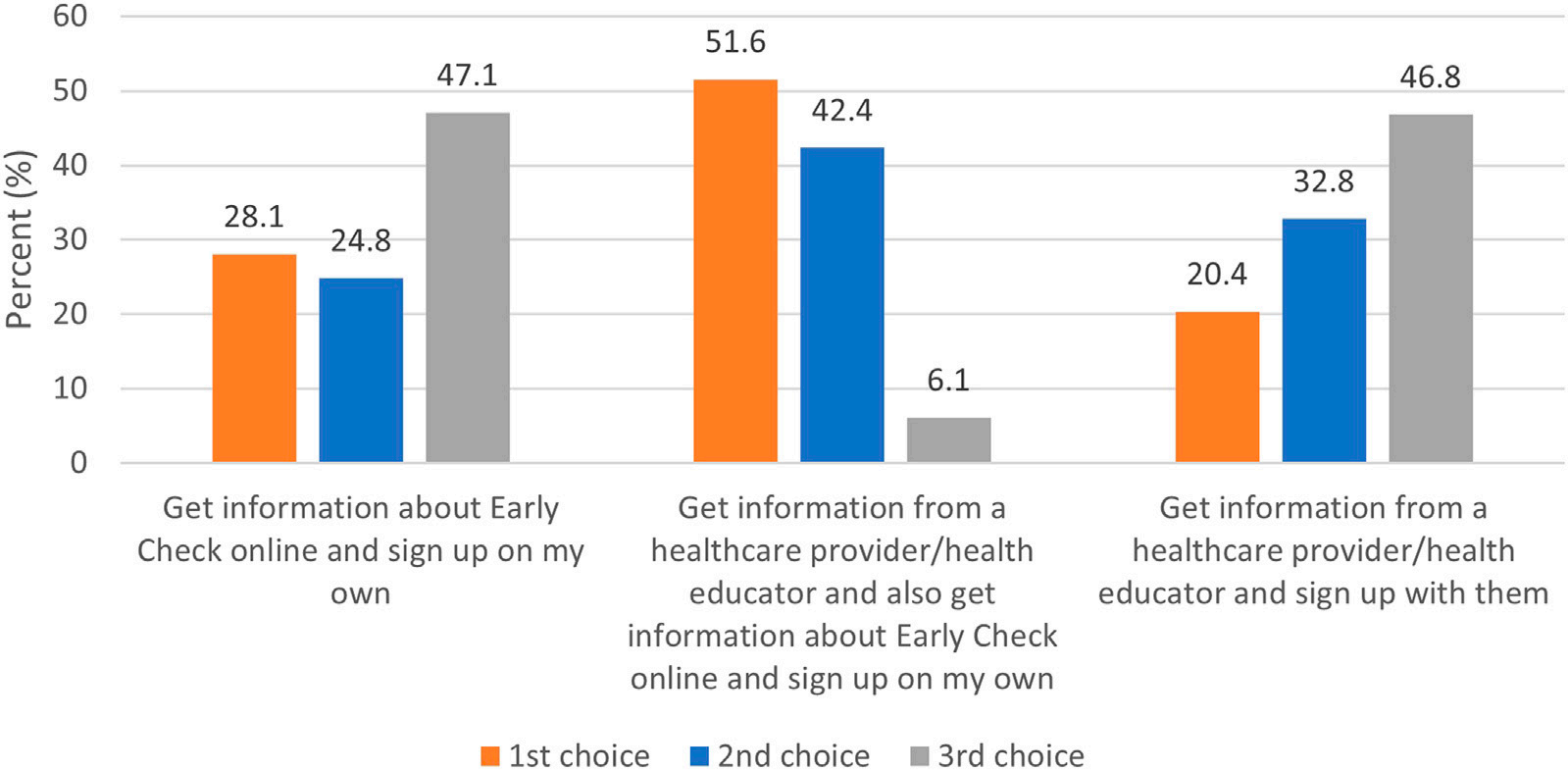
Ranking of preference for education and consent (*n* = 1,542).

When asked about preferences for getting information about and enrolling in Early Check, the most preferred option was to get information from a healthcare provider and from Early Check online, and sign up on my own (51.6%), followed by get information about Early Check online and sign up on my own (28.1%). The least-preferred option was to sign up with a healthcare provider (20.4%) (Figure 2).

**FIGURE 3:**
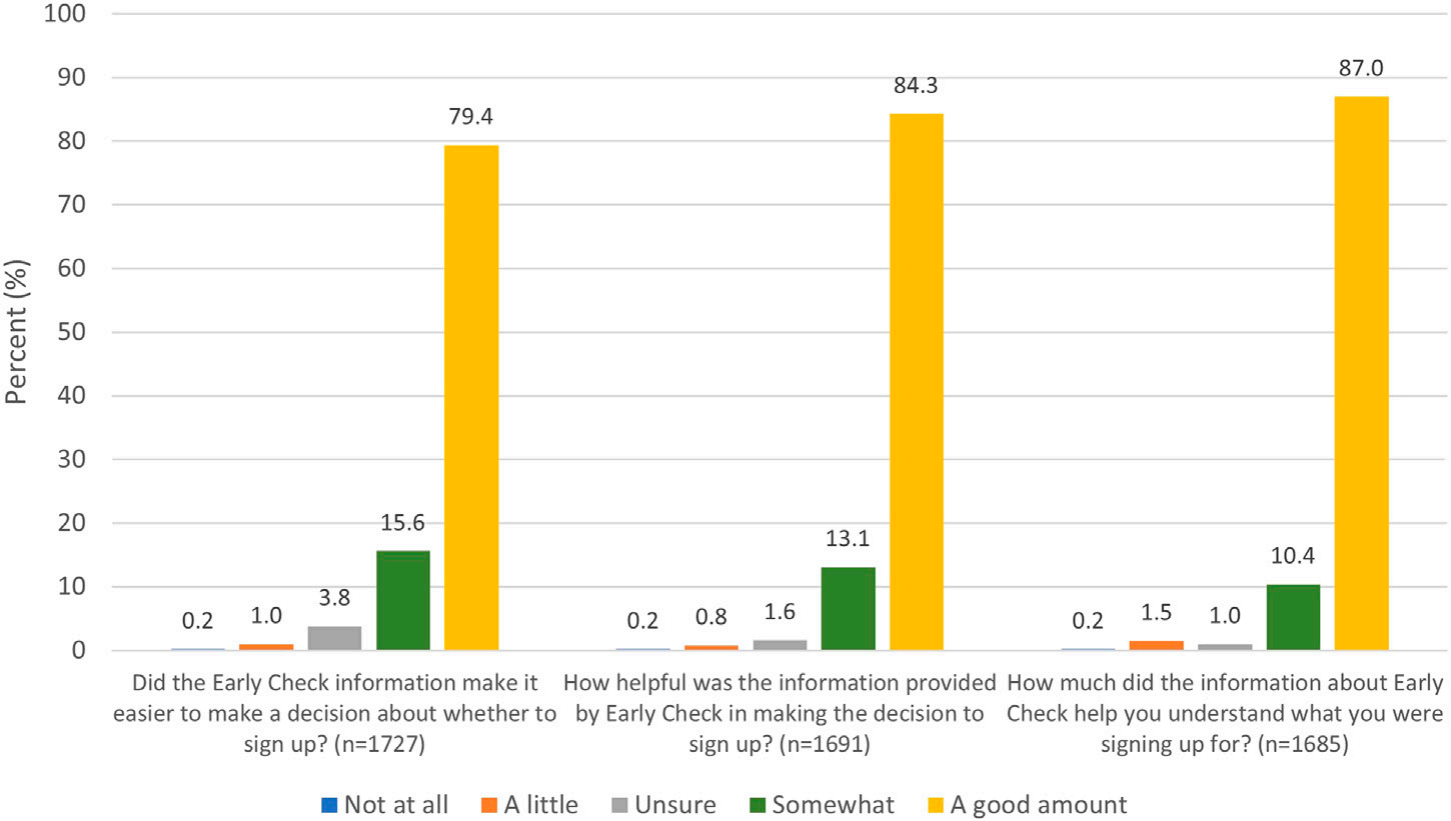
Perceived utility of study information to decision making.

On a scale of 0–5, survey respondents reported that the Early Check information made it easier to decide whether to sign up (mean = 4.73), was helpful in making the decision (mean = 4.81), and helped them understand what they were signing up for (mean = 4.83) (Figure 3).

Most survey respondents (98.2%) reported that they received enough information about Early Check, and 99.1% indicated that other parents would be able to decide with the same information (Table 3).

**TABLE 3:** Information sufficiency (*n* = 1,708).

	All	
	N	%
Did you get enough information about Early Check?		
No	31	1.8
Yes	1,677	98.2
With the same information you got, do you think other parents will be able to make a decision about signing up for Early Check?		
No	15	0.9
Yes	1,693	99.1

Those who indicated that they did not get enough information (*n* = 31, 1.8%) were asked what else they hoped to learn (Table 4.) The most common response was to learn more about the conditions screened (*n* = 22), followed by the Early Check process and standard newborn screening (*n* = 12).

**TABLE 4:** What respondents who felt they did not get enough information about Early Check hoped to learn (*n* = 31).

	N
More about the conditions screened	22
More about the Early Check process	12
More about newborn screening	12
More about the child’s participation and expectations	9
Other	4

#### 3.3.2 Interviews

All but one interviewee reported that it was easy to sign up; that respondent reported that it was neither easy nor difficult. Ease of enrollment was described as a motivating factor for most respondents. Reasons for perceiving the enrollment process as easy included: information that was easy to understand, an entirely online enrollment process, no need for additional information from parents to sign up (e.g., from medical records), and that it did not take long to sign up.

“Yeah, the fact that it was really easy to do. It was just like: ‘Oh, just click here, click here.’ If I were to go on the page and it would have been confusing or messy […] I would not have clearly been shown how to sign up, I’m sure that I would not have done [it]. But it was so easy that I just was like ‘click, click’, you know?”

When asked to describe how they felt when visiting the Early Check website, the most common response was feeling more informed. Several described the content as “straightforward” and that they did not have many questions after viewing the portal.

“I did not have a lot of questions about it. I thought, ‘why would anybody not do this?’ And I remember it wasn’t challenging. It was just do X, Y, and Z.”

Interview respondents were asked whether they received enough information to sign up, whether the information was clear and complete, and if they understood which conditions were screened. Most responded in the affirmative to these questions. Respondents were asked whether there was any information not included on the website that they would have wanted. Most said no information was missing and they did not need to search for more information beyond what was provided. Two respondents had to look elsewhere for information on whether the screening was only available for newborns (or if it was also available for older children) and the conditions screened in standard newborn screening.

### 3.4 Attitudes About Early Check Screening and Participation

#### 3.4.1 Evaluation Survey

Attitudes about the screening were positive among survey respondents. Mean scores on the attitude items, measured on a scale of 0–4 with lower scores indicating better attitudes, are shown in Table 5. Survey respondents reported that Early Check screening was “important” (0.58), “desirable” (0.32), “reassuring” (0.18), a “good thing” (0.12), and “beneficial” (0.17).

**TABLE 5:** Attitudes about screening.

For me, having early check screening was	N (%)					Mean (SD)	
	0	1	2	3	4				
Beneficial	620	57	23	5	1	Harmful	0.17 (0.52)
	(87.82%)	(8.07%)	(3.26%)	(0.71%)	(0.14%)		
Important	436	149	102	16	2	Unimportant	0.58 (0.84)
	(61.84%)	(21.13%)	(14.47%)	(2.27%)	(0.28%)		
A good thing	641	43	19	2	0	A bad thing	0.12 (0.42)
	(90.92%)	(6.10%)	(2.70%)	(0.28%)	(0.00%)		
Reassuring	614	54	31	3	0	Not reassuring	0.18 (0.51)
	(87.46%)	(7.69%)	(4.42%)	(0.43%)	(0.00%)		
Desirable	539	106	50	4	2	Undesirable	0.32 (0.66)
	(76.89%)	15.12%)	(7.13%)	(0.57%)	(0.29%)		

In responses to the three items selected from the Decision Regret Scale (Brehaut et al., 2003), 98.6% strongly agreed or agreed that participation was the right decision; 96.7% strongly disagreed or disagreed with regretting participation; and 99.3% strongly agreed or agreed that they would make the same choice again (Table 6).

**TABLE 6:** Decision regret for Early Check participation.

	Frequency	Percent
It was the right decision		
Strongly agree	564	80.6
Agree	126	18.0
Neither agree nor disagree	9	1.3
Strongly disagree	1	0.1
Frequency missing = 93		
I regret the choice that was made		
Strongly agree	12	1.7
Agree	5	0.7
Neither agree nor disagree	6	0.9
Disagree	87	12.5
Strongly disagree	585	84.2
Frequency missing = 98		
I would go for the same choice if I had to do it over again		
Strongly agree	598	85.4
Agree	97	13.9
Neither agree nor disagree	3	0.4
Strongly disagree	2	0.3
Frequency missing = 93		

#### 3.4.2 Interviews

Interviewees indicated high satisfaction with participation. All stated that they would sign up if given the chance to make the decision over again, for reasons that were similar to their motivations for enrollment: ease of participating, being armed with the information about their child, and contributing to research. Further, nearly all stated that they would recommend Early Check to a friend; the one respondent who would not recommend it indicated that she would not think to do so.

### 3.5 Knowledge Recall About Early Check

#### 3.5.1 Evaluation Survey

**FIGURE 4:**
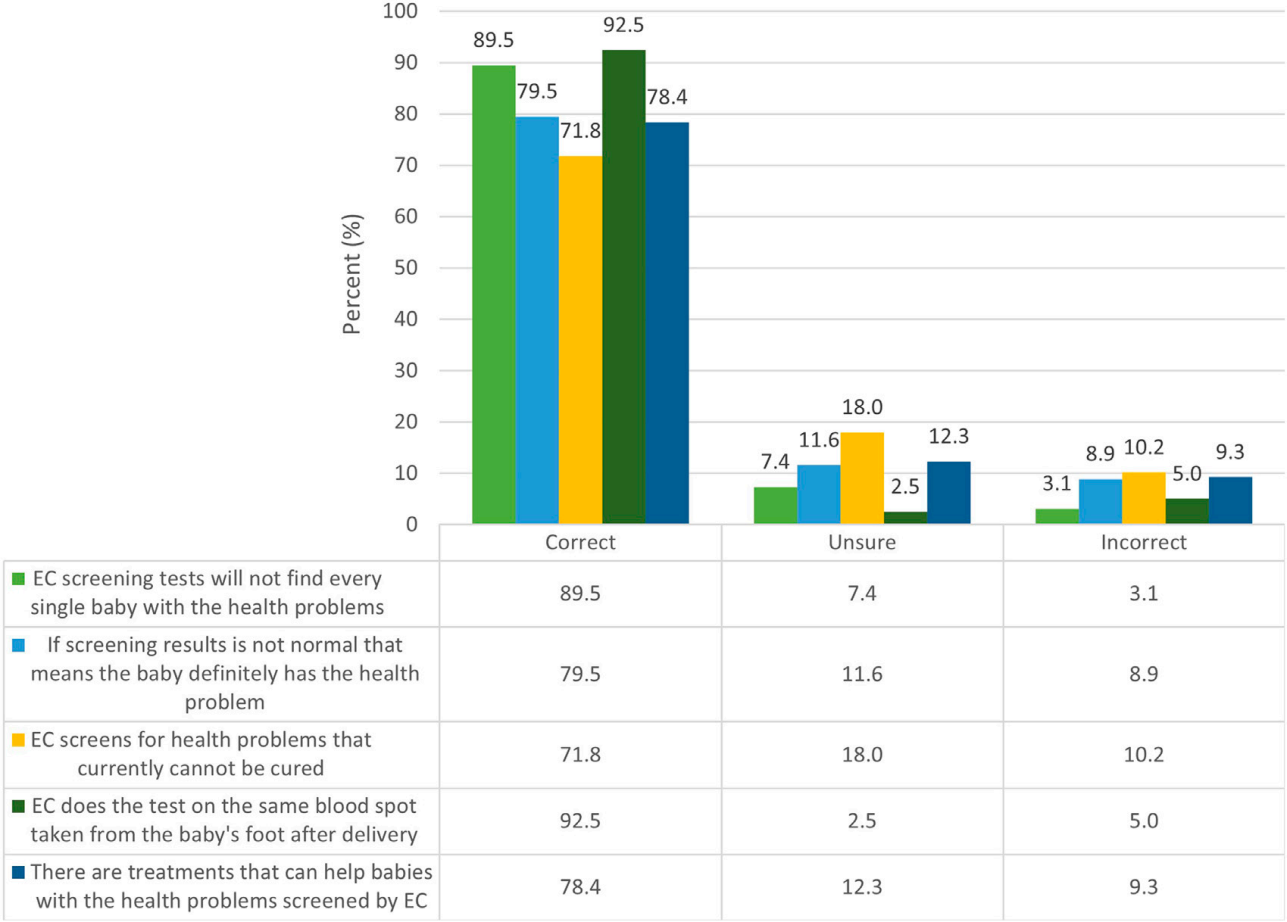
Recall of key Early Check concepts (*n* = 1,630).

Most survey respondents correctly recalled key concepts from the electronic consent materials. A large majority (92.5%) correctly recalled that Early Check performs the test on the same blood spot taken from the baby’s foot after delivery and 89.5% that the screening tests will not find every baby with the health problems. Most (78.4%) correctly identified that there are treatments that can help identified babies; but that Early Check screens for health problems that currently cannot be cured (71.8% correct); and 79.5% correctly identified as false the concept that an abnormal result means the baby definitely has the health problem (Figure 4).

Using a summed knowledge recall score, an unadjusted, ordered logistic regression was used to determine whether there were significant differences based on mothers’ age, between White and non-White participants, between Hispanic/Latino and non-Hispanic/Latino participants, and among those with less than a bachelor’s degree, a bachelor’s degree, or more than a bachelor’s degree. Maternal age was not significant in the unadjusted model and thus was not included in the adjusted model. An adjusted model with all significant characteristics found that, similar to the adjusted model (Table 7), White, non-Hispanic, and more-highly-educated respondents were more likely to score higher on knowledge recall.

**TABLE 7:** Ordered logistic regression: Knowledge recall score^a^.

*n* = 1,346	Unadjusted		Adjusted	
	OR^b^	95% CI	OR^b^	95% CI
Race				
White	5.74***	(4.03, 8.17)	4.0***	(2.78, 5.76)
Non-White (ref.)				
Ethnicity				
Non-Hispanic	2.14***	(1.57, 2.92)	1.63**	(1.13, 2.33)
Hispanic (ref.)				
Education				
< Bachelor’s degree	0.40***	(0.31, 0.52)	0.45***	(0.34, 0.59)
Bachelor’s degree (ref.)				
> Bachelor’s degree	1.63***	(1.31, 2.0)	1.64***	(1.13, 2.33)

**p < *0.01*.

***p < *.0001.*

a Knowledge recall score is the sum of the number of recall questions answered correctly. Range is 0–6.

b OR (Odds Ratio) greater than one means the participant characteristic is positively associated with a higher knowledge recall score, and a less than one means the characteristic is negatively associated with a knowledge recall score.

#### 3.5.2 Interviews

All interviewees agreed that the information on the Early Check website was clear and complete, but most did not remember any specific information or sections of the consent content. Those who did remember specifics most often reported remembering the video elements on the website.

“I think the video is easier to understand and I think some people don’t have the patience to read all those words and they prefer the video. I think though the video is good for that kind of parent…”

### 3.6 Trust in the Information Provided About the Early Check Study

#### 3.6.1 Evaluation Survey

**FIGURE 5:**
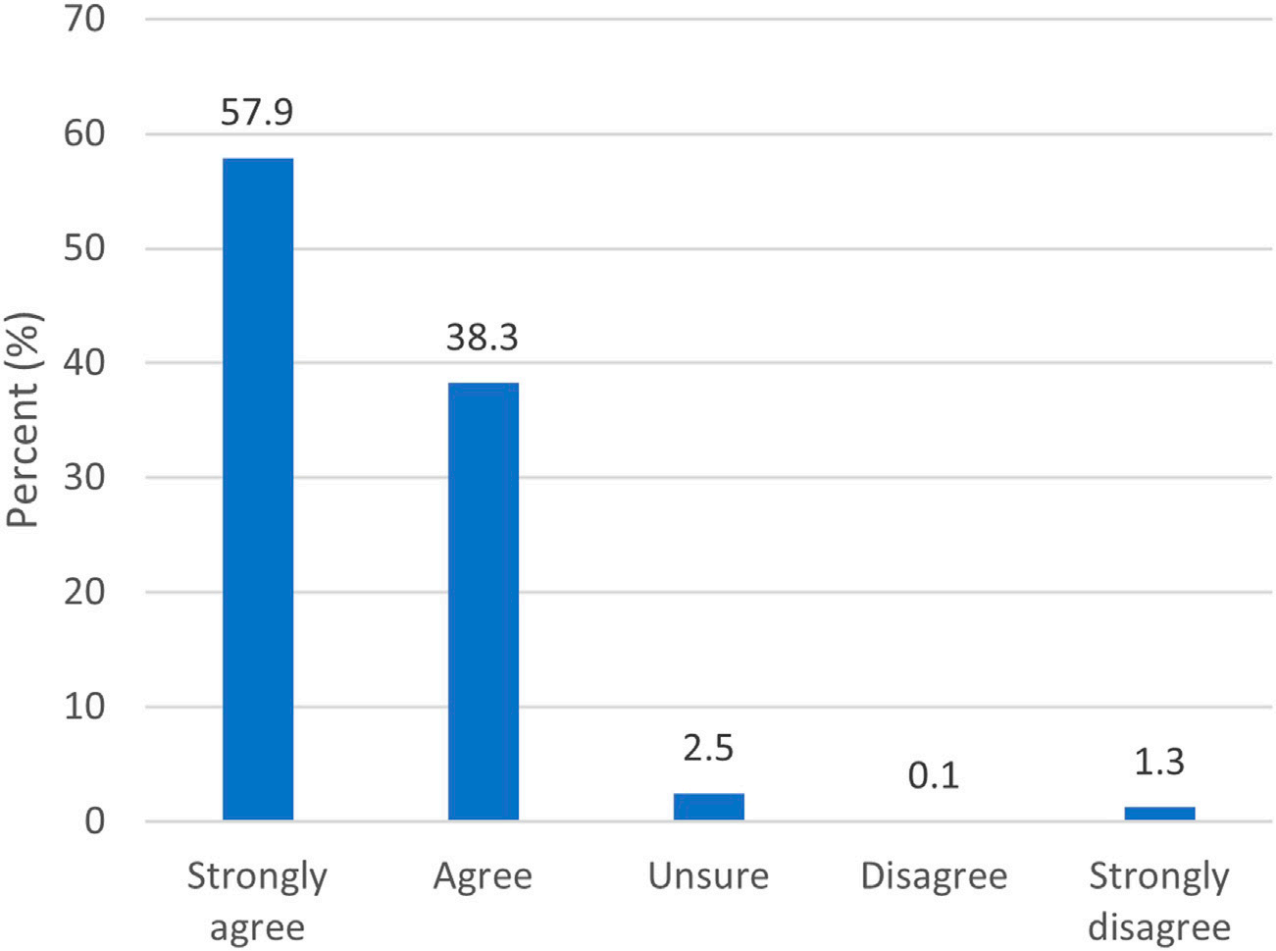
Responses for “I trust the information provided by Early Check” (*n* = 1,661)

Most survey participants reported that they trusted the information provided by Early Check, with 57.9% selecting “strongly agree” and 38.3% selecting “agree” (Figure 5).

**FIGURE 6:**
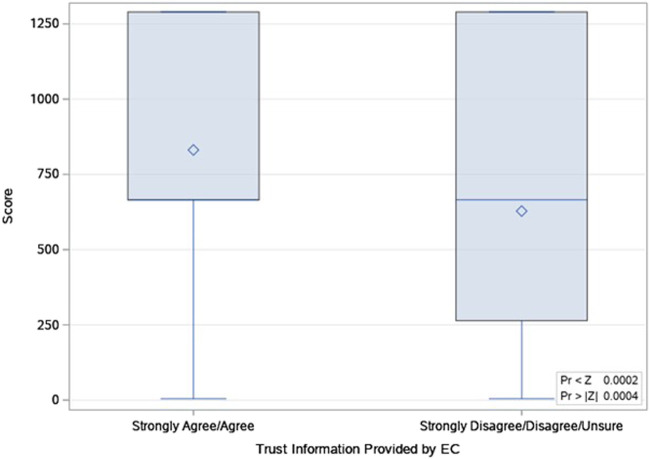
Distribution of Wilcoxon Scores for knowledge score, by trust in Early Check.

In assessing those who reported trust (*n* = 1,598) versus those who indicated being unsure or distrusting Early Check (*n* = 63), there were significant differences based on race and education. In addition, those reporting less trust were significantly more likely to report more negative attitudes toward the screening (*p* < 0.001) and to indicate that they were not sufficiently informed (*p* = <0.0001). The mean knowledge recall score is higher for those who trust the information versus those who do not (Z = -3.51, *p* < 0.001) (Table 8 and Figure 6.)

**TABLE 8:** Factors associated with trust in Early Check participants.

	Trust (*n* = 1,598)	Unsure/Distrust (*n* = 63)	*p*-value
Race			**0.007**
White	1,208 (79.68%)	38 (64.41%)	
Non-White	274 (18.07%)	17 (28.81%)	
Prefer not to say	34 (2.24%)	4 (6.78%)	
Education			**0.039**
<Bachelor’s degree	351 (22.99%)	22 (37.29%)	
Bachelor’s degree	482 (31.57%)	15 (25.42%)	
>Bachelor’s degree	694 (45.45%)	22 (37.29%)	
Attitude about screening [Mean (SD)]	1.39 (2.27)	2.70 (3.20)	**0.0002**
Knowledge recall score	4.12 (1.00)	3.54 (1.38)	**0.0005**
Informed enough			**< 0.0001**
Yes	1,576 (98.62%)	57 (90.48%)	
No	22 (1.38%)	6 (9.52%)	

Bold values indicate *p*-value from Chi-Square or Fisher’s exact test for categorical, Kruskal-Wallis test for ordinal variables, and Mann-Whitney U test for interval data.

#### 3.6.2 Interviews

All interviewees reported that they trusted the information provided by Early Check. Many said that the information was from a credible source and the website appeared legitimate. Several also noted that the organizations listed on the website made them trust the information, and most participants said that they were familiar with at least one of the institutions.

“Yeah…the fact that you’re doing surveys on it, it looked like a lot of thought went into planning, how it was laid out and how it was worded. That even if I wasn’t good at using a website, or even if I wasn’t good at reading, what seemed very scientific or medical, I could still understand it. It seemed like there was care put into it to make it seem not intimidating and intentional and well-worded and stuff.”

“I mean as far as like you, the schools of Wake Forest and UNC and Duke, I mean, all those are, you know, I recognize that they’re all like research organizations and local universities. So, I thought that they seemed reputable. It wasn’t like here were a random company trying to collect your child’s genetic information.”

## 4 Discussion

We developed a large-scale education and consent approach that was designed to be feasible for the study team, acceptable and trustworthy to parents making decisions about enrollment, and promoting of informed decisions. During our 16-month evaluation period we enrolled over 7,700 infants to Early Check, the large majority coming through our entirely participant-driven online education and consent process. We have demonstrated that our participant-driven, online approach makes it feasible to educate a large sample from the general population.

And yet developing an approach, that is, feasible for the study team only has utility if it also meets the needs of the end users. This requires developing study materials that provide sufficient information while maintaining a reasonable and acceptable level of complexity and literacy. Our survey respondents reported that the study information was sufficient and made it easier to make an enrollment decision and understand what they were signing up for. These sentiments were echoed by parents who participated in the qualitative interviews, who expressed that information was easy to understand, easy to navigate, and informative for decision making. Existing literature on the use of electronic consent is also promising with studies reporting positive attitudes and experiences of participants who use virtual approaches informed consent (Bollschweiler et al., 2008; Abujarad et al., 2018; Simon et al., 2018).

Our data indicate that our participant-driven, online approach was acceptable to those who agreed to participate. Survey respondents most preferred an approach that included healthcare provider and online information, with online sign up; this was followed by online only. Survey respondents and interviewees reported positive attitudes and limited regret about their decision to enroll their newborns. Ease and convenience were cited as motivations to enroll, which is a common-sense finding. Study teams can, however, make it too easy to enroll. It is well-recognized that online users are accustomed to scrolling through content to get to the accept button without reading technical information (Doerr et al., 2016). The process of education for screening and consent for research participation must not take advantage of that learned behavior. It may be important for content and interface developers to build “friction” into the online education process; this includes purposefully-designed elements to slow and engage users (Doerr et al., 2016). Employing a variety of media may meet this goal while also offering different approaches to learning that do not rely solely on reading (Rowbotham et al., 2013; Kraft et al., 2017; Simon et al., 2018). In our website materials we employed voiceover, simple graphics, infographics, video, brief values clarification, and self-assessment questions. We designed the user interface to promote exposure to the core content and required participants to click through content rather than scrolling.

Most survey respondents correctly recalled key concepts of Early Check, similar to the evaluation of the All of Us research program’s electronic consent (Doerr et al., 2021). Interviewees were not asked equivalent questions where specific concepts were assessed due to the exploratory nature of the interviews. Therefore, it is unclear whether interview participants recall these concepts similarly. Our survey data indicate areas for improvement in explaining educational concepts—particularly the differentiation between treatment and cure. Although the overall numbers are small, we acknowledge that our knowledge recall is lower in non-White populations and those with less education. It is paramount that population-focused programs continue efforts to develop education, that is, effective for those from diverse racial, ethnic, and education backgrounds.

Another critical goal of Early Check is trustworthiness. Regardless of the quality of educational materials, some degree of trust is required for parents to agree to enroll their child in screening. We found high trust in Early Check; our qualitative data indicate that having sufficient information and clearly identifying collaborating institutions, especially those known through the state, is important. Among the fewer than 4% of survey respondents who indicated distrust or being unsure about trusting the information, we observed more individuals identifying in race categories other than white, less positive attitudes toward Early Check, and lower information recall. Although our materials include multiple references to the voluntary nature of participation and brief values clarification component that reviews why parents may choose to decline Early Check participation for their children, parents who are unsure or untrusting of Early Check may still anticipate sufficient value from the resulting screening information to offset feelings of distrust.

A strength of our study is that we obtained both quantitative and qualitative data. The interviews allowed us to explore unexpected findings that would not have emerged from a survey. Although results from the interviews and surveys were complementary, the survey questions and the qualitative interview questions were not identical.

### 4.1 Limitations

A limitation to our data is that our evaluation participants have higher education than the average in the state of North Carolina. About 30% of the North Carolina working-age population has a bachelor’s degree or higher (U.S. Census, 2020) compared to 74% in our evaluation survey respondents. As such, our findings have limited generalizability. In addition, we achieved only a 24% response rate in our survey. The relatively low response rate may be to some extent explained by a study team decision to de-emphasize the evaluation survey in favor of promoting communication about the return of screening results; clearly it is more important to garner the attention of participants to their newborn’s screening result than to recruit for the evaluation. Further, our data may be biased based on time between enrollment and data collection (recall bias) and social desirability bias. To help reduce the potential for bias in the qualitative data collection and interpretation, we employed an interviewer who was not involved in the day-to-day operations of Early Check and analysts who were completely uninvolved with the Early Check study prior to coding the data.

It should be noted that this evaluation comprised parents who received negative (or normal) screening results. Parents who received positive screening results may have differing views. We are conducting additional research on mothers of children who screen positive to explore the impact of the positive screen and their experiences and attitudes, and their recommendations for improving Early Check procedures. Another important limitation is that this study included only mothers who enrolled their children in Early Check and not those who declined participation. The study population must be taken into account when interpreting our findings, as these are individuals who perceived Early Check to be sufficiently trustworthy and the screening of sufficient value to warrant participation. Additional research on those who do not participate in Early Check, although ethically and practically challenging, is important to informing population-based DNA screening.

### 4.2 Implications

Large-scale research and public health use of DNA-based screening become increasingly feasible when quality electronic approaches are used to educate and/or consent impacted communities. Our evaluation of the Early Check newborn screening research study indicates that participant-focused materials provided in an entirely virtual format can be acceptable, trustworthy, and informative. Though developing participant-focused materials is a time-intensive process that requires a multidisciplinary development group and the use of community engagement and formative research, the result can be a user-directed process that requires little study team time. Early Check currently uses single-gene and analyte screening; we are in process of adapting and testing a similar approach for newborn screening using exome sequencing, where some educational concepts are of higher complexity. Additional evaluation data from programs that use virtual education and consent may lead to best practices in new material development and may increase the acceptance of participant-centered electronic consent among regulators. Finally, as DNA-based screening programs and screening-based studies are implemented, it is vital to explore new approaches to education and consent that account for the needs of diverse target populations.

## Data Availability

The raw data supporting the conclusions of this article will be made available by the authors, if the data request is in accordance with the IRB protocol and consent.
